# SNP in Potentially Defunct Tetrahydrocannabinolic Acid Synthase Is a Marker for Cannabigerolic Acid Dominance in *Cannabis sativa* L.

**DOI:** 10.3390/genes12020228

**Published:** 2021-02-04

**Authors:** Andrea R. Garfinkel, Matthew Otten, Seth Crawford

**Affiliations:** Oregon CBD, Independence, OR 97351, USA; mjotten@jackhempicine.com (M.O.); seth@jackhempicine.com (S.C.)

**Keywords:** cannabinoids, cannabinoid synthesis, CBG, hemp

## Abstract

The regulation of cannabinoid synthesis in *Cannabis sativa* is of increasing research interest as restrictions around the globe loosen to allow the plant’s legal cultivation. Of the major cannabinoids, the regulation of cannabigerolic acid (CBGA) production is the least understood. The purpose of this study was to elucidate the inheritance of CBGA dominance in *C. sativa* and describe a marker related to this chemotype. We produced two crossing populations, one between a CBGA dominant cultivar and a tetrahydrocannabinolic acid (THCA) dominant cultivar, and one between a CBGA dominant cultivar and a cannabidiolic acid (CBDA) cultivar. Chemical and genotyping analyses confirmed that CBGA dominance is inherited as a single recessive gene, potentially governed by a non-functioning allelic variant of the THCA synthase. The “null” THCAS synthase contains a single nucleotide polymorphism (SNP) that may render the synthase unable to convert CBGA to THCA leading to the accumulation of CBGA. This SNP can be reliably used as a molecular marker for CBGA dominance in the selection and breeding of *C. sativa*.

## 1. Introduction

*Cannabis sativa* L. is an herbaceous annual grown for its wide variety of uses including for fiber and feed, and for its use in producing compounds with therapeutic and psychoactive properties. Recent lifting of restrictions on the cultivation and sale of *C. sativa* in the United States has led to a proliferation of research into the use of this crop in pharmaceuticals.

*C. sativa* produces organic molecules known as “cannabinoids” or “phytocannabinoids,” of which nearly 150 have been identified [1]. These cannabinoids are of specific interest for their demonstrated value in nearly all realms of medicine from the treatment of sleep disorders [2], to epilepsy [3], to cancer [4].

Not all *C. sativa* plants produce all of the known cannabinoids in equal ratios. Plants of *C. sativa* have been grouped into five broad categories, or “chemotypes,” based on the relative ratios of three predominant cannabinoids: tetrahydrocannabinolic acid (THCA), cannabidiolic acid (CBDA), and cannabigerolic acid (CBGA) [5,6]. So-called “Type I” plants comprise of individuals that contain predominantly THCA, and contain low levels of CBDA; “Type II” plants contain THCA and CBDA in approximately equal ratios; “Type III” plants have high levels of CBDA, and relatively low levels of THCA; “Type IV” plants contain CBGA, which is also the precursor compound to both CBDA [7] and THCA [8], as the predominant cannabinoid; “Type V” plants are characterized by undetectable amounts of any of these three cannabinoids and are referred to as “cannabinoid-free” [5,6].

The inheritance of these chemotypes have been well studied in a series of papers by de Meijer and co-authors [5,9,10,11,12]. In these papers, the authors proposed that THCA to CBDA content ratios in Types I, II, and III plants were regulated by a two-allele system characterized by distinct forms at a single locus: one form responsible for THCA production (referred to as B_T_) and the other CBDA production (referred to as B_D_) [5,9,10,11,12]. In this single-locus model, Type I plants contained a homozygous B_T_:B_T_ genotype, Type II plants contained a heterozygous B_T_:B_D_ genotype, and Type III plants the homozygous B_D_:B_D_ genotype [5]. Genetic mapping and molecular cloning [13], followed later by whole genome sequencing of *C. sativa* showed that these two alleles are not at the exact same physical location as proposed, but are situated at two distinct loci on the same chromosome and are most likely linked [14]. Therefore, these genes are inherited in a fashion like the single-locus model proposed by de Meijer et al. [5]. In the plants studied by whole genome sequencing, the THCA-dominant strain assessed contained a tetrahydrocannabinol acid synthase (THCAS) and only a pseudogenic, non-functional copy of a cannabidiol acid synthase (CBDAS), whereas the CBDA-dominant plant contained a CBDAS, but no apparent copies, pseudogenic or otherwise, of a THCAS in the expected location [14].

In their model, de Meijer and Hammond [9] proposed that Type IV plants contained a third, null allele B_0_ that is biochemically unable to convert CBGA into THCA or CBDA, thus leading to CBGA accumulation. While de Meijer and Hammond [9] did not provide any sequence data to confirm their null locus model, they hypothesized that the null allele was a sequence variant or mutation of the B_D_ allele. Onofri et al. [15] followed up with gene sequencing and found that out of the three CBGA-dominant cultivars, two contained copies of a gene with sequence homology to the CBDAS and one contained a gene with sequence homology to the THCAS. Onofri et al. [15] compared the gene sequences from their Type IV plants with “wild-type” alleles from Type I and Type III plants and concluded that the alleles in Type IV plants displayed unique single nucleotide polymorphism (SNP) patterns. Therefore, the authors proposed that these sequence variants represented null alleles and were given the names B_DO_, sequence variant of B_D_ of which there were two types, and B_T0_, sequence variant of B_T_ [15].

Although Onofri et al. [15] suspected the role of these null alleles in CBG accumulation, we do not know of any studies that have carried out crosses to confirm the inheritance of these putative null cannabinoid synthase alleles and their associations with resultant chemotypes. In this paper, we present data from Type IV cultivars containing a novel THCAS which may represent an additional B_T0_ allele sequence variant and provide evidence for its inheritance and its role as a marker in CBGA dominance.

## 2. Materials and Methods

### 2.1. Plant Material

All CBG cultivars used in the trials herein were developed as a result of the self-pollination of a plant received via an order through Seedsman (Barcelona, Spain). The initial controlled cross was performed by E. Crawford in 2016 in Eugene, OR, USA. The original plant had been determined to have trace CBGA content as based on HPLC analyses (performed by OG Analytical, Eugene, OR, USA). A total of five seeds were harvested as a result of self-pollination and plants were grown up and tested for their cannabinoid composition. A total of two of the five plants were determined to be Type III and were destroyed; three plants, named TS1-2, TS1-3, and TS1-5, were determined to be Type IV and were saved. TS1-3 and CBGA-dominant cultivar FB30 were used in trials. FB30 was developed as a result of a selective breeding process (described in next section) using TS1-3.

### 2.2. Breeding Crosses

FB30 was developed using TS1-3 as the Type IV parent in the initial F1 cross. A series of open pollinations and outcrosses resulted in a single individual, FB30, that was then selfed; a single individual from the selfed population was used in the cross described below. Throughout the breeding process for Type IV parents, mendelian recessive inheritance of the CBGA chemotype reported by de Meijer and Hammond [9] was observed. All crosses and selections were performed at Oregon CBD (Independence, OR). CBDA- and THCA-dominant plants used in test crosses were also developed by the same company or used under license.

A total of two segregating populations were developed for genotype and chemotype analysis in order to test the hypothesis that the novel THCAS sequence variant is a marker for CBGA dominance. For the first population, “Cross TE”, TS1-3 (Type IV) was crossed to a Type III plant, ERB, and a single individual was then selected to produce a selfed F_2_ population, resulting in a population of 102 individuals. For the second population, “Cross FH”, a single selfed FB30 individual (Type IV) was pollinated by a Type I plant, HO40. A single individual was then selected to produce a selfed F_2_ population and then a single heterozygous (B_T_:B_T0_) individual (plant #11) was then selected to produce a selfed F_3_(S_2_) population; the resultant 105 progeny were screened. All plants were genetically female (contained two X chromosomes), but one of the parents in each cross was treated three times with 750 ppm silver thiosulphate solution at five-day intervals to induce male flower production for pollination [16,17].

Segregation ratios for chemotypes were calculated for each cross and observed and expected values were compared using a Pearson’s chi-square test for independence.

### 2.3. Chemotyping

Chemical analyses were performed according to a protocol modified from Vaclavik et al. [18]. Tissue samples for analysis were collected from the first fully expanded leaf following the development of alternating phyllotaxis, which is an indication of sexual maturity in *C. sativa* plants [19]. Wellington et al. [19] demonstrated that chemical compositions of leaf samples from sexually mature *C. sativa* plants correspond to those found in flower tissue and were, therefore, considered an accurate representation of chemotype in the current study. Each sample was prepared by adding 1–2 g of plant material to a 50 mL polypropylene Falcon tube which contained a 6 mm stainless steel grinding ball (SPEX, cat. no. 2154), and frozen at −80 °C for at least 1 h. Plant material was pulverized (SPEX 1600 MiniG Automated Tissue Homogenizer) at 1150 rpm for 50 s.

Cannabinoids were extracted in the same Falcon tube with the addition of 30 mL HPLC grade methanol and vortexed on a Cole-Parmer Multi-TubeVortexer at 2500 rpm for 30 min. The extracted sample was then filtered through a 13 mm 0.2 µm PFTE Acrodisk cartridge (Pall 4423T) into an HPLC sample vial with a PTFE septa cap. Extractions not analyzed the same day were stored at −80 °C until use.

Plant samples were analyzed for cannabinoid content by High Performance Liquid Chromatography (HPLC) using an Agilent 1260 Infinity II with diode array detector (DAD) with OpenLab CDS ChemStation software. A Restek Raptor ARC-18, 150 × 4.6 mm × 2.7 µm reverse phase column with Raptor ARC-18, 5 × 4.6 × 2.7 µm guard column was utilized under the following operating conditions: 1.8 mL/min flow rate, 30.0 °C column temperature, 228 nm wavelength, 4 nm bandwidth, 9 min run time, and 1 min post run. The mobile phase gradient is 65→90% mobile phase B over 6.5 min., then 2.5 min. hold, with mobile phase A: 0.1% formic acid in water and mobile phase B: 0.1% formic acid in acetonitrile.

Cannabinoid standards for CBDA (CAS No. 1244-48-2), CBGA (CAS No. 25555-57-1) and Δ^9^-Tetrahydrocannabinolic acid (Δ^9^-THCA-A) (CAS No. 23978-85-0) at 1000 µg/mL were obtained from Cerilliant Corporation (Round Rock, TX, USA). These were used to determine retention times of each cannabinoid and to prepare a 7-point calibration curve from 1→100 µg/mL. A calibration verification standard was injected at the start of each analysis day to verify retention times and quantitation.

All peaks ≥3x signal to noise were integrated and a percent area report was generated. The relative ratio between cannabinoids was calculated by dividing the higher percentage value by the lower percentage value. Therefore, CBDA (74.90):Δ^9^-THCA-A (2.73) = 27.44, or a 27.4:1 ratio (see examples in Tables S1–S6). Representative chromatograms from samples from the TE population are included as Supplementary Materials (Figures S1–S6). Ratios were used in lieu of total cannabinoid (TC) content due to the need for the completion of flowering to perform analyses, which was not possible due to current legal limitations of working with Type I cultivars (included in the FB crossing populations) in the United States where the study was conducted. Welling et al. [19] demonstrated that leaf tissue samples taken from sexually mature *C. sativa* plants in the vegetative phase directly corresponds to cannabinoid proportions in terminal flowers and Weiblen et al. [13] found that TC was independent of major cannabinoid ratios. Therefore, although not critical to the results of our study, we have reason to believe the final flower ratios would be similar to those reported herein. For the FH population, THCA and THCVA (tetrahydrocannabivarinic acid) and CBGA and CBGVA (cannabigerovarinic acid) were pooled to calculate the THCA(V):CBGA(V) ratio as the parent HO40 was known to be high in the propyl variant of THCA and CBGA, respectively. A similar strategy was used by Onofri et al. [15]. Propyl paralogs were not included in the calculations for the TE population because the samples were not evaluated for CBGVA due to the prior knowledge that only negligible quantities existed in parents and F1 progeny. The calculated ratios for the different genotypes were compared using an analysis of variance conducted in SAS University Edition (SAS Institute Inc., Cary, NC). Means separations were conducted using Fisher’s least square means with an α of 0.05.

### 2.4. Nucleic Acid Extractions

DNA extractions for PCR and qPCR were executed using a *Quick*-DNA Plant/Seed Miniprep Kit or a *Quick*-DNA Plant/Seed 96 Kit (Zymo Research, Irvine, CA, USA) according to the manufacturer’s instructions, except that samples were initially homogenized after being frozen at −80 °C followed by a second homogenization step using the BashingBead^TM^ buffer included with the kit. Shoot tips without fully expanded leaves were used in all DNA extractions.

RNA extractions were performed using a *Quick*-RNA Miniprep Kit (Zymo Research, Irvine, CA, USA) including the optional DNA digest step as per the manufacturer’s instructions. Samples for RNA extraction were taken from female flowers during their receptive stage (containing white stigmas) for all samples except the Type 1 plants from which leaf tissue was taken due to aforementioned legal limitations in working with THCA(V)-dominant flower material. All samples were harvested and immediately frozen in liquid nitrogen.

### 2.5. Cannabinoid Acid Synthase PCR, qPCR, RT-qPCR, and Sequencing

Primers “a” and “b” from Kojoma et al. [20] were used in PCR to amplify the THCAS from CBGA-dominant cultivars TS1-3 and FB30 and THCA(V)-dominant cultivar HO40 in 20 μL reactions containing: 1X PCR reaction buffer (Genscript), 0.2 μM of each dNTPs (Genscript), 0.25 μM of each primer (IDT, Coralville, IA, USA), 3 U Taq DNA Polymerase (Genscript), and variable amounts of DNA template. PCR amplification conditions were: 94 °C for 5 min; 35 rounds of 94 °C for 30 s, 52 °C for 30 s, and 72 °C for 75 s; followed by a final extension at 72 °C for 5 min. PCR products were visualized using a FlashGel system (Lonza) and then cleaned up using Exo-SAP IT Express (Affymetrix). Primers “a,” “b,” “d,” “e,” and “f” from Kojoma et al. [20] were used in sequencing (Genewiz). Raw sequence chromatograms were assessed for quality and accurate base pair calling and then edited sequences were aligned, and consensus sequences were built using Geneious Prime v. 2019.2.3. Several other THC-dominant cultivars were sequenced using the same primers and protocol to determine additional allelic variants (see results).

Primers CBDAS_1F and CBDAS_1R were developed to amplify the CBDAS from Type III plant ERB. Reaction conditions were the same as those reported above for PCR and sequencing of the THCAS. The same primers, plus an additional primer, CBDAS_a_F, were used in sequencing. Primer sequences are reported in Table 1. Type III cultivar ERB was also subjected to sequencing using the THCAS primers described above to ensure that no THCAS was present in this cultivar.

A multiplex qPCR assay to detect the presence or absence of the THCAS and CBDAS alleles was developed. Primers and probe sequences were as reported in Table 1. All primers and probes were ordered and synthesized by IDT. Probes were fluorescently labeled on the 5′ end with FAM or HEX fluorophores and synthesized with internal ZEN and 3′ Iowa Black^®^ FQ quenchers (IDT). Primers and probes to amplify the THCAS and CBDAS were designed to amplify all sequence variants of these genes as reported in GenBank, including the putative “null” THCAS allele reported by Onofri et al. [15] and the THCAS allele later reported in this paper to be associated with our CBGA-dominant cultivars; special care was taken to design the THCAS primers to avoid cross-amplification with the cannabichromenic acid synthase (CBCAS), to which the THCAS shares high sequence homology [15]. Reactions were performed in 15 μL volumes containing 1X TaqMan Fast Advanced Master Mix (Thermo Fisher Scientific), 7.5 nM of each of the primers CBDAS_6F, CBDAS_6R, THCAS_2F, and THCAS_2R, 3.75nM of each of the probes CBDAS_6P and THCAS_2P, and variable amounts of template DNA. qPCR was performed on a QuantStudio 5 (ThermoFisher) in 0.2 mL, 96-well qPCR plates using the “Standard Curve” and “Fast” run options with the following conditions: 50 °C for 2 min, 95 °C for 2 min, and then 40 cycles of 95 °C for 1 s and 60 °C for 20 s.

The same primer set used for the THCAS in the qPCR multiplex detection assay were also used in detecting cDNA in RNA expression analyses. RNA expression analyses were performed in 15 uL, one-step reactions containing: 1X TaqMan Fast Virus 1-Step Master Mix (ThermoFisher) 7.5 nM of each primer, 3.75 nM of probe, water, and 2 uL of template RNA (variable concentrations). qPCR was performed on a QuantStudio 5 (ThermoFisher) in 0.2 mL, 96-well qPCR plates using the “Standard Curve” and “Standard” run options with the following conditions: 50 °C for 5 min, 95 °C for 20 s, and then 40 cycles of 95 °C for 15 s and 60 °C for 60 s.

A Custom TaqMan SNP Genotyping Assay (ThermoFisher) was designed to detect a single nucleotide polymorphism observed in the THCAS of Type IV plants TS1-3 and FB30 (see results for additional information on SNP identity). The TaqMan SNP Genotyping Assay contained forward primer ANAAKA9_F (5′ ACTGATTGCAAAGAATTTAGCTGGATTG 3′), reverse primer ANAAKA9_R (5′ CAAGCAAAATTTCCTTTTTAAAATTAGCAGTGT 3′), and probes ANAAKA9_V (5′/VIC/CCATCTTCTACAATGGTGTT/MGB NFQ/3′) and ANAAKA9_M (5′/FAM/CATCTTCTACAGTGGTGTT/MGB NFQ/3′) (the location of the 1064 bp SNP is indicated in the probe sequences in bold lettering). SNP genotyping assays were performed in 10 μL reactions using the “Fast” genotyping cycling conditions using TaqPath ProAmp Master Mix (ThermoFisher) according to the manufacturer’s protocol. Calls were made automatically by the genotyping software, however, were manually checked for call accuracy. In all analyses, TS1-3 was run as a homozygous control for the null THCAS allele (hereafter also referred to as THCAS_0_), HO40 was run as the homozygous control for the wild type (non-null) THCAS allele (hereafter also referred to as Wt THCAS or THCAS_Wt_), and a known heterozygote developed as the result of an F_1_ cross between the two cultivars was used as a heterozygous control.

Alignments of cannabinoid synthase genes with all primers and probes used in this study are reported in Figures S7 and S8.

## 3. Results

### 3.1. Cannabinoid Synthase Sequence Identity

Following PCR, sequencing, and alignment, a full-length consensus sequence of the THCAS was obtained from cultivars TS1-3, FB30, and HO40. The sequences from TS1-3 and FB30 shared 100% sequence homology over the 1635 intronless gene region (not including the stop codon). The sequence of TS1-3 was used to search in the NCBI GenBank database for similar sequences using the MEGABlast search engine; no identical sequences were identified (BLAST results from 9 June 2020). The sequences with the closest sequence identity were from C. sativa clones ABC35 and ABC7 (accession numbers KT876015 and KT875987, respectively), both of which were identical except for a single SNP as compared with TS1-3 and FB30 at 1064 bp (Table 2). The chemotype of clones ABC35 and ABC7 could not be confirmed. The sequences of TS1-3 and FB30 were also compared to additional sequences in GenBank and it was determined that this SNP was unique compared to all the C. sativa THCAS entries in GenBank. Furthermore, this SNP was different from the putative null THCAS (B_T0_) observed in the Type IV plant reported by Onofri et al. [15] (Table 2). Two additional Type I cultivars sequenced in this study, Cake Breath and Animal Cookies (chemotype data upon request), were determined to have identical sequences to TS1-3 and FB30 THCAS sequence variant except for the SNP located at position 1064 (Table 2). The THCAS sequences from Cake Breath and Animal Cookies were submitted to GenBank (accession numbers MW504063 and MW504064, respectively).

The SNP at 1064 bp resulted in an amino acid change from serine to asparagine at position 355 (Table 2). TS1-3 and FB30 shared an additional SNP at bp position 998 with C. sativa clones ABC35 and ABC7, which resulted in an amino acid change at position 333 as compared with other selected gene sequences collected from GenBank (Table 2). The THCAS gene sequence of TS1-3 was submitted to GenBank (accession number MT338560).

Sequencing revealed that the THCAS from the Type I plant HO40 contained the most common base pair at every site among the THCAS sequences selected from GenBank (Table 2). The THCAS sequence from HO40 was submitted to GenBank (accession number MW382908).

Sequence analysis also confirmed the presence of two allelic variants of a CBDA synthase in Type III cultivar ERB. The CBDAS sequence (with both allelic variants represented using ambiguity codes for the relevant nucleotides) from ERB was submitted to GenBank (accession number MW382907). PCR primers a and b, and sequencing primers a, b, d, e, and f [20], produced a gene sequence with 99.9% sequence homology to a CBCAS reported by Page and Stout [21], as expected for non-THCA-dominant plants which contain this gene [15]. The CBCAS from ERB was submitted to GenBank (accession number MW504065).

### 3.2. Heritability of the Type IV Chemotype

SNP genotyping indicated that CBGA-dominance was found to be consistently related to the presence of the homozygous state of the THCAS sequence variant containing the G to A SNP at base pair position 1064 in both populations. The presence of the THCAS containing the mutation in the homozygous state predicted the Type IV chemotype with 100% accuracy.

#### 3.2.1. Cross TE

**Figure 1:**
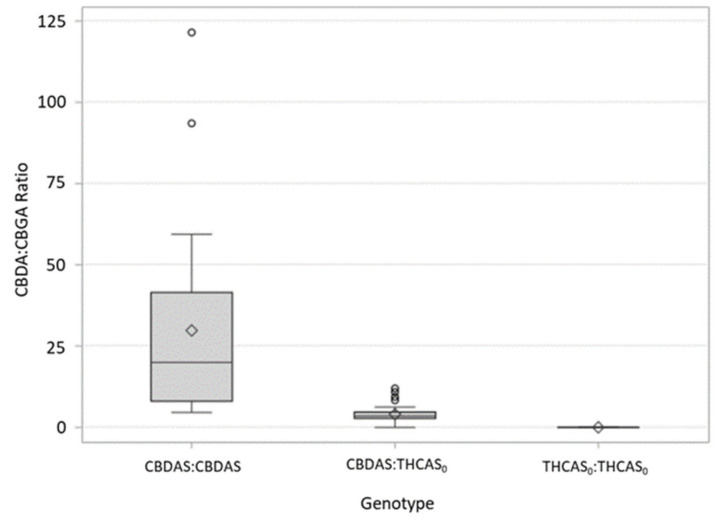
Distribution of CBDA:CBGA ratios in Cross TE, a backcrossed F_2_ population of 102 individuals resulting from an F_1_ cross between a Type III (CBDA-dominant) plant and a Type IV (CBGA-dominant) *C. sativa* plant. The arithmetic mean is indicated with a diamond. Whiskers indicate 1.5 × the inner quartile range and open circles are observations that fall outside of this parameter.

Cross TE resulted in a total of 102 progeny that were genotyped and chemotyped. qPCR analysis of the progeny showed that 23 (22.5%) individuals were homozygous for the CBDAS, 56 (55%) were heterozygous and contained both a CBDAS and a THCAS allele, and 23 (22.5%) were homozygous for the THCAS. These segregation ratios fit the expected 1:2:1 model of single-locus inheritance of these alleles (*p* = 0.61). PCR and sequencing of the THCAS was carried out on an additional 24 individuals using the protocol described above; all plants with the CBG chemotype were confirmed to contain the THCAS allele containing the G to A SNP at base pair position 1064 in the homozygous state. HPLC data were generated for all of the 102 individuals from Cross TE. CBDA:CBGA ratios ranged from 0.008 to 121.5, with Type III (CBDAS:CBDAS) plants having the highest average ratio of 29.9 and Type II (CBDAS:THCAS_0_) and Type IV (THCAS_0_:THCAS_0_) having statistically lower average ratios than Type III plants (*p* =< 0.0001) of 4.07 and 0.02, respectively. Cannabinoid ratios between Type II and Type IV plants did not statistically differ from one another (Figure 1 and Table 3).

#### 3.2.2. Cross FH

Cross FH resulted in a total of 105 progeny. The SNP analysis suggested that 30 (28.6%) plants were homozygous for the Wt THCAS allele, 56 (53.3%) plants were heterozygous and contained both the Wt THCAS allele and the putatively defunct null THCAS allele, and 16 (15.2%) were homozygous for the null THCAS allele; three plants (2.8%) could not be definitively genotyped using the SNP qPCR assay. The three individuals that could not be genotyped using the SNP assay were subject to sanger sequencing of the THCAS as described in the methods and all were determined to be homozygous for the putatively null THCAS allele, bringing the total count of homozygotes to 19 (18.1%). Of the 105 plants that were genotyped, the progeny met the expected 1:2:1 segregation ratio (*p* = 0.25).

**Figure 2:**
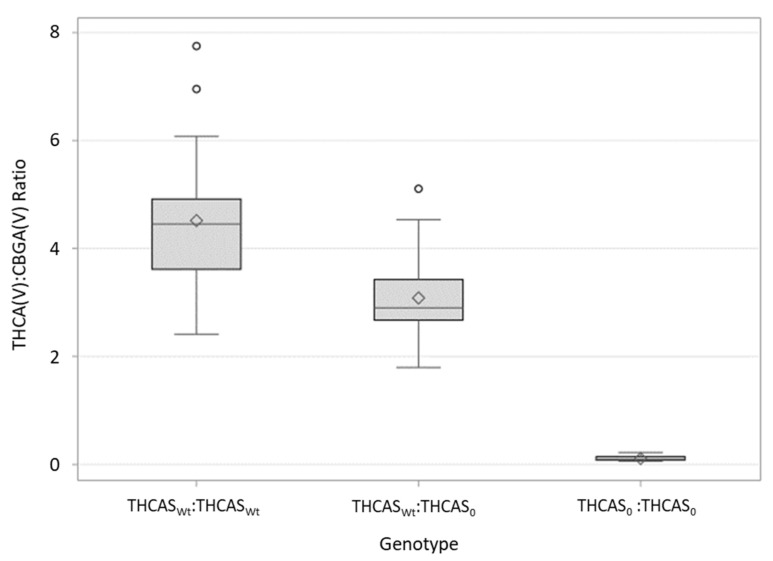
Distribution of THCA(V):CBGA(V) ratios in Cross FH, a selfed F_2_ population of 99 individuals resulting from an F_1_ cross between a Type I (THCA(V)-dominant) plant and a Type IV (CBGA-dominant) *C. sativa* plant. The arithmetic mean is indicated with a diamond. Whiskers indicate 1.5 × the inner quartile range and open circles are observations that fall outside of this parameter.

Chemotyping was performed on a subset of plants due to poor plant health and the inability to get sufficient tissue for analysis from some of the individuals. Of the 105 plants that had been genotyped, chemotype data were obtained for 99. Similar to what was seen with Cross TE, there were significant differences between individuals containing various copy numbers of the novel THCAS containing the G1,064A mutation (*p* =< 0.0001). The THCA:CBGA ratio was significantly different (α = 0.05) among all three genotypes; those plants which SNP genotyping indicated as heterozygotes containing one copy of the Wt THCAS and one of the novel THCAS had an average THCA(V):CBGA(V) ratio of 3.1, those containing two copies of the Wt THCAS allele had an average ratio of 4.5, and plants containing two copies of the novel THCAS allele had an average THCA(V):CBGA(V) ratio of 0.1 (Figure 2).

### 3.3. Gene Expression

Expression of the putatively null THCAS was confirmed in the flowers of Type IV plant TS1-3 as assayed by RT-qPCR using the THCAS probe and primer set. Additional Type I and Type III plants were used as controls; similar amplification was seen in Type I plants and no amplification was observed in the Type III plants. Tissue samples were highly heterogenous among samples for RNA extraction and no internal control gene was used in expression analyses; therefore, only presence or absence of expression is reported. A cycle threshold (Ct) value of 35 was used as the cutoff to determine presence or absence of gene expression. Type I plants and Type IV plants consistently amplified products before cycle 35. Template from Type III plants failed to amplify prior to cycle 35.

## 4. Discussion

The allele observed in our CBGA-dominant cultivars has not been identified previously according to our search of the literature and current GenBank entries and is to our knowledge the second sequence variant of the THCAS that has been associated with Type IV plants [15]. This sequence variant of the THCAS is consistently and predictably present in the homozygous state in *C. sativa* plants which contain CBGA as the dominant cannabinoid, as shown by crosses with both THCA(V)- and CBDA-dominant plants. Our crosses also confirm the recessive mendelian inheritance of CBGA-dominance as first reported by de Meijer and Hammond [9]. As per current hypotheses regarding the regulation of cannabinoid dominance in *C. sativa*, it is possible that this SNP and resultant amino acid change is responsible for the “inactivation” of the THCAS, leading to its inability to effectively convert the precursor molecule CBGA into THCA. Further analyses involving the expression and ability of this enzyme to convert CBGA into THCA in vitro would be required to confirm this hypothesis.

Unusually, the SNP associated with the Type IV plants in this study do not correspond to any of the other known SNPs suspected to alter the function of the THCAS. In addition to the putative defunct THCAS reported by Onofri et al. [15] with which the present allele does not share sequence homology, several other studies have investigated the structural variations of the THCAS protein and tested its conversion activity in the presence of several mutations. Mutant enzymes containing single amino acid changes have been shown to result in the reduced activity or complete inactivation of the THCAS [20,21,22]. However, none of the previously tested changes that have been shown to inactivate the THCAS include the S355N amino acid change observed in our Type IV plants. The regions of the THCAS protein previously shown to reduce or eliminate THCAS activity include mutations to a flavin adenine dinucleotide (FAD) binding domain [22,23], several gycolization sites, and a berberine bridge enzyme domain [24]. The amino acid change observed in the present Type IV cultivars does not appear in any of these regions as identified by Sirikantaramas et al. [22] and Shoyama et al. [23].

Plants that were genotyped as heterozygous, either containing one copy of the null THCAS allele and a CBDAS allele, or those containing one copy of the null allele and one Wt THCAS allele, showed intermediate chemotypes, in which higher amounts of CBGA were present than in their homozygous counterparts not containing a null THCAS allele. Although the average CBD:CBG ratios were not statistically significant between Type II and Type IV plants in Cross TE, the ratios are biologically meaningful and suggest that a single copy of the CBDAS present in the Type II plants is converting small amounts of CBGA to CBDA, albeit at a lower efficiency than if two copies were present. Together, the data from Crosses 1 and 2 suggest that when present only as a single copy in the genome, the CBDA and THCA synthases have a limited ability to convert CBGA, yet have additive effects when present as two copies. de Meijer and Hammond [9] noticed a similar pattern and speculated that the rate of CBG accumulation is greater than the conversion rate of the cannabinoid synthases. Although heterozygotes in both the Type I and Type III crosses had higher proportions of CBGA than the homozygotes lacking the null allele, they were, nonetheless, largely predominant in either THCA or CBDA, respectively. The presence of intermediate chemotypes, yet with obvious either THCA- or CBDA-dominance, was also reported by de Meijer and Hammond [9]. The ratio of predominant cannabinoid (THCA or CBDA) to CBGA varied among individuals and crosses (Figure 1 and Figure 2), suggesting that there are other genes involved in regulating cannabinoid production not explained or explored in the current study. Interestingly, the effect of heterozygosity (having one copy of the “null” THCAS and a single copy of a fully-functional cannabinoid synthase allele) appears to be different among the crosses. Heterozygous individuals in the FH cross appear to be more efficient in their conversion of CBGA to THCA(V) than heterozygotes in the TE are in their conversion of CBGA to CBDA (Figure 1 and Figure 2). Although noteworthy, it is perhaps not surprising as different cannabinoid synthase sequence variants have been shown to affect cannabinoid composition, including the ratios of THCA(V):CBGA(V) and CBDA:CBGA [15]. It is possible also that the putatively null sequence variant has a negative regulatory effect on the fully-functional CBDAS, but not the fully-functional THCAS. Indeed, there are likely several factors regulating cannabinoid content ratios that are not addressed in this paper and deserve further analysis. This discrepancy could also be a result of selecting the F1 for different traits; the F1 in the FH population was selected for low THCA(V) content whereas the F1 in the TE population was selected for high CBDA content (Table 3 and Table 4). These results would also suggest the presence of additional genes/loci responsible for determining cannabinoid ratios.

In this manuscript, we have referred to the heterozygous plants containing one copy of a CBDAS and one copy of a null THCAS “Type II” according to previous nomenclature established by de Meijer et al. [5] to describe plants with the one copy of a THCAS and one copy of a CBDAS. However, since the THCAS present in our described “Type II” plants is not of an intermediate CBDA:THCA chemotype, this designation is not completely accurate from a chemical phenotypic standpoint. Given that there are multiple null alleles which appear to be responsible for CBGA-dominance [15], not all of which are THCAS homologs, it may perhaps be appropriate to designate additional *C. sativa* types which would represent plants that contain one fully-functioning major cannabinoid synthase gene (a THCAS or CBDAS) and one null allele of either the THCAS or CBDAS type, and are therefore an intermediate CBGA chemotype. Theoretically, these additional *C. sativa* variants would represent four genotypic combinations; the cannabinoid ratios among the different genotypes would have to be further explored to understand the relationship between the different combinations of allelic variants. Furthermore, the traditional B_D_:B_T_ allele model may also require modification with the discovery of additional B_0_ variants and, perhaps more importantly, in light of the findings that these two genes are not true allelic variants insofar as they are not physically located at the same location in the genome [14].

Despite having dramatically reduced levels of THCA, plants that contain both null THCAS alleles still contain small quantities of THCA, with the amount varying among individuals and crossing populations (Figures S3 and S6, Tables S3 and S6). It is possible that, while leading to a reduction in the conversion of CBGA to THCA, the mutant allele is still able to convert small amounts of the precursor molecule. This could potentially explain why, despite the apparent reduced conversion of CBGA to THCA, our qPCR analyses suggest that the THCAS is expressed in CBGA-dominant plants. Onofri et al. [15] also found that the putative defunct THCAS in their CBGA-dominant plant was expressed at relatively high levels, in some cases higher in comparison with expression levels in Type I plants. Alternatively, it is possible that another cannabinoid synthase gene is responsible for the THCA seen in the Type IV plants, of which there are many in the genome (although notably, we have produced whole genome sequence data, that is not shown as it is outside of the scope of this manuscript, which indicate that our Type IV parental lines lack a CBDAS or CBCAS homolog), or that the mutation is not truly leading to an inactivation of the THCAS, but simply serves as a marker for a linked region which is involved in producing the Type IV chemotype and silencing THCA production by a separate mechanism. Although it is yet unconfirmed that the THCAS gene we report is indeed unable to convert CBGA to THCA, our results indicate that it is a useful marker for breeding for CBGA-dominance in *C. sativa* in populations containing this mutation.

## Figures and Tables

**Table 1:** Primer and probe sequences used for qPCR in this study to amplify cannabinoid synthase genes in *Cannabis sativa*.

Target Gene	Primer/Probe Name	Direction	Sequence (5′ to 3′)
CBDAS	CBDAS_1F	Forward	TTTATTTGCCCCTGCTCCAA
	CBDAS_1R	Reverse	TGGAGCATACACAGTACATCCG
	CBDA_a_F	Sequencing	GGAGCTACCCTTGGAGAAGTT
CBDAS	CBDAS_6F	Forward	CTTTGGTGGAGGAGGCTATG
	CBDAS_6R	Reverse	TCCATGAACGTTGACTAAGTGT
	CBDAS_6P	Probe	/56-FAM/TGATGAGAA/ZEN/ACTATGGCCTCGCGG/3IABkFQ/
THCAS	THCAS_2F	Forward	CCCTCATCGAGCTGGAATAAT
	THCAS_2R	Reverse	GGGACACATAAGGAGTCGTAAA
	THCAS_2P	Probe	/56-HEX/ACTTTGGTA/ZEN/CACTGCTTCCTGGGA/3IABkFQ/

**Table 2:** Single nucleotide polymorphisms among tetrahydrocannabinol acid synthase (THCAS) genes from selected cultivars.

		Nucleotide position ^2^											
**Acc. No. ^1^**	**Chemotype**	187	366	373	399	706	749	998	1064	1229	1232	1395	1563
**TS1-3**	IV	A	A	G	A	G	C	**G**	**A**	G	A	T	G
**FB30**	IV	A	A	G	A	G	C	**G**	**A**	G	A	T	G
KP970850	IV	A	A	G	A	**C**	C	C	G	G	A	T	G
**HO40**	II	A	A	G	A	G	C	C	G	G	A	T	G
**Cake Breath**	I	A	A	G	A	G	C	**G**	G	G	A	T	G
**Animal Cookies**	I	A	A	G	A	G	C	**G**	G	G	A	T	G
AB212838	II	A	A	G	A	G	C	C	G	G	A	T	G
AB212829	II	**C**	**T**	G	**G**	G	C	C	G	G	A	T	G
AB212832	II	A	A	G	A	G	C	C	G	G	A	T	G
AB212834	II	A	A	G	A	G	C	C	G	G	A	T	G
AB212835	II	A	A	G	A	G	C	C	G	G	A	T	G
AB212837	II	A	A	G	A	G	C	C	G	G	A	T	G
AB212838	II	A	A	G	A	G	C	C	G	G	A	T	G
KT875984	II	A	A	G	A	G	C	C	G	G	A	T	G
KT875981	II	A	A	G	A	G	**A**	C	G	G	A	T	**A**
KT875987	II	A	A	G	A	G	C	**G**	G	G	A	T	G
KT875985	II	A	**T**	**C**	A	G	C	C	G	**A**	A	**A**	G
KT875988	II	A	A	G	A	G	C	C	G	G	A	T	G
KT875994	II	A	A	G	A	G	**A**	C	G	G	A	T	G
KT875990	II	A	A	G	A	G	C	C	G	G	A	T	G
KT875998	II	A	A	G	A	G	**A**	C	G	G	A	T	G
KT876005	II	A	A	G	A	G	C	C	G	G	A	T	**A**
KT876015	II	A	A	G	A	G	C	**G**	G	G	A	T	G
KT876006	II	A	**T**	**C**	A	G	C	C	G	**A**	A	**A**	G
KT876046	II	A	A	G	A	G	C	C	G	G	**C**	T	G
KT875999	II	A	A	G	A	G	C	C	G	G	A	T	G
		**Amino Acid Position ^3^**											
		**63**		**125**		**236**	**250**	**333**	**355**	**410**	**411**		
		I→L		V→L		E→Q	A→D	P→R	S→N	G→E	A→V		

^1^ Names in bold represent cultivars used in this study. Accession numbers beginning with “KP” reported from Onofri et al. [15], accession numbers beginning with “AB” are from Kojoma et al. [20], and accession numbers beginning with “KT” are unpublished. ^2^ The base pairs highlighted in gray are putatively responsible for inactivation of the THCAS leading to the accumulation of cannabigerolic acid. ^3^ Amino acid positions and changes are indicated in the rows at the bottom of the table. Where no amino acid is shown, a change does not occur.

**Table 3:** Mean CBDA:CBGA ratios among three genotypes tested in Cross TE and the CBDA:CBGA ratios of parents and F1 progeny.

Genotype/Parent	n ^1^	Mean CBDA:CBGA Ratio ^2^	Standard Deviation
CBDAS:CBDAS	23	29.8 a	29.91
CBDAS:THCAS_0_	56	2.12 b	2.13
THCAS_0_: THCAS_0_	23	0.02 b	0.007
ERB	-	189.84	-
TS1-3	-	0.02	-
F1	-	14.43	-

^1^ Chi-squared test statistics for 1:2:1 expected segregation ratio: df = 2; χ^2^ = 0.98; *p*-value = 0.61. ^2^ Analysis of variance test statistics: df = 2; Type III SS = 13,172.71; F-value = 32.73; *p*-value =< 0.0001. Means followed by the same letter are not statistically different (α = 0.05). Numbers not followed by letters are not means, but actual ratios for the single individual reported. A dash (-) indicates data are not applicable.

**Table 4:** Mean THCA(V):CBGA(V) ratios among three genotypes tested in Cross FH.

Genotype	n ^1^	Mean THCA(V):CBGA(V) Ratio ^2^	Standard Deviation
THCAS_Wt_:THCAS_Wt_	30	4.52 a	1.2
THCAS_Wt_:THCAS_0_	56	3.08 b	0.66
THCAS_0_: THCAS_0_	19	0.11 c	0.04
HO40	-	39.61	-
FB30	-	0.01	-
F1	-	4.15	-

^1^ Chi-squared test statistics for 1:2:1 expected segregation ratio: df = 2; χ^2^ = 2.77; *p*-value = 0.25. ^2^ Analysis of variance test statistics: df = 2; Type III SS = 217.56; F-value = 175.94; *p*-value =< 0.0001. Means followed by the same letter are not statistically different (α = 0.05). Numbers not followed by letters are not means, but actual ratios for the single individual reported. A dash (-) indicates data are not applicable.

## Data Availability

The sequence data presented in this study are openly available in GenBank; accession numbers are given in the text. Additional chemotyping data may be available upon request to the authors.

## Supplementary Materials

The following are available online at https://www.mdpi.com/2073-4425/12/2/228/s1, Figures S1–S6: Representative sample chromatograms from each genotype of the TE and FH populations; Figures S7 and S8: Alignments of select cannabinoid synthase with primers and probes used in this study; Tables S1–S6: Representative sample percent area reports for assessing cannabinoid content in *C. sativa* for each genotype from the TE and FH populations.
