# Supplementary material for: SNP in Potentially Defunct Tetrahydrocannabinolic Acid Synthase Is a Marker for Cannabigerolic Acid Dominance in Cannabis sativa L
Source: Genes (Basel). 2021 Feb 4;12(2):228. doi: 10.3390/genes12020228 (PMC7916091; doi:10.3390/genes12020228)
Supplement: Supplementary file 1 [file genes-12-00228-s001.pdf]

Supplementary information for Garfinkel et al. 2021

SNP in potentially defunct tetrahydrocannabinolic acid synthase is a marker for cannabigerolic acid dominance in *Cannabis sativa* L.

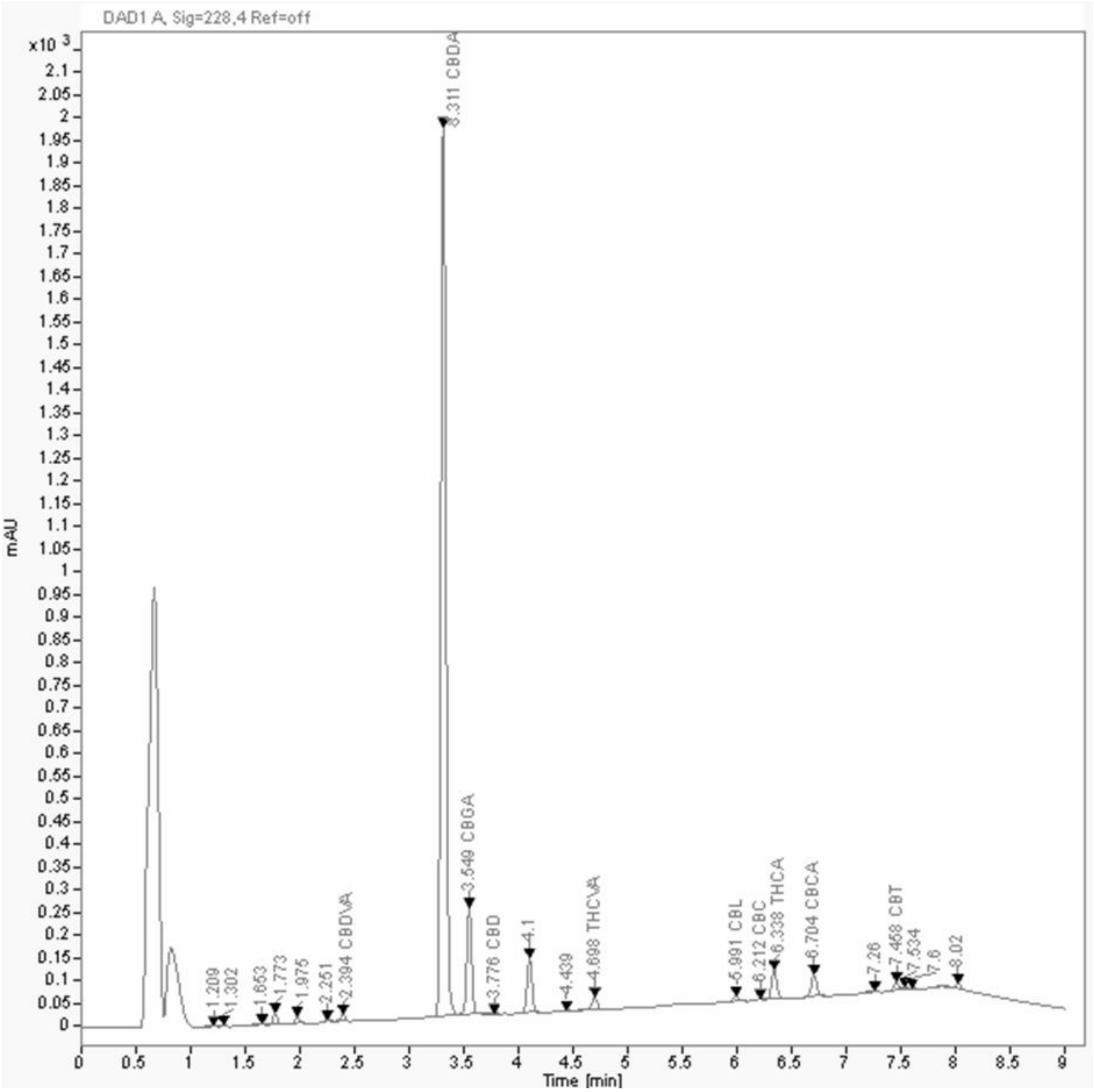

Figure S1. Chromatogram for assessing cannabinoid content in *C. sativa* from individual #4 from the TE population that has a CBDAS:CBDAS genotype.

Table S1. Percent area report for assessing cannabinoid content in *C. sativa* from individual #4 from the TE population that has a CBDAS:CBDAS genotype.

Signal:		DAD1 A, Sig=228,4 Ref=off				
RT [min]	Type[1]	Width [min]	Area	Height	Area%	Name[1]
1.209	BV	0.0465	6.0250	2.0390	0.0726	
1.302	VB	0.0403	7.5025	2.8909	0.0904	
1.653	BB	0.0637	11.3640	2.5645	0.1369	
1.773	BB	0.0464	75.7625	25.6861	0.9129	
1.975	BB	0.0472	44.8533	15.7429	0.5405	
2.251	MM	0.0441	12.0375	4.5498	0.1450	
2.394	MM	0.0460	43.7774	15.8763	0.5275	CBDVA
3.311	BV	0.0487	6216.3330	1978.1511	74.9044	CBDA
3.549	VV R	0.0494	766.7592	239.0426	9.2391	CBGA
3.776	BB	0.0538	3.4925	0.9744	0.0421	CBD
4.100	BB	0.0502	375.9146	120.9976	4.5296	
4.439	BV	0.0458	3.7665	1.3008	0.0454	
4.698	BB	0.0540	94.5344	27.5675	1.1391	THCVA
5.991	BB	0.0628	50.8471	12.6818	0.6127	CBL
6.212	BB	0.0473	9.9169	3.2814	0.1195	CBC
6.338	BB	0.0544	226.3036	65.4115	2.7269	THCA
6.704	BB	0.0553	174.9766	49.4510	2.1084	CBCA
7.260	MM	0.0570	9.0612	2.6494	0.1092	
7.458	BV	0.0495	64.7363	20.1197	0.7800	CBT
7.534	VV	0.0473	19.7715	6.1843	0.2382	
7.600	VB	0.0384	4.0202	1.5423	0.0484	
8.020	MM	0.1039	77.2703	12.3973	0.9311	
		Sum	2502.1845			

[1]Indicates type of integration. BV = baseline-to-valley; VB = valley-to-baseline; BB = baseline-to-baseline; MM = manually integrated; R = re-calculated solvent.

[2]Name of cannabinoid. CBDVA = cannabidivarinic acid; CBDA = cannabidiolic acid; CBGA = cannabigerolic acid; CBD = cannabidiol; THCVA = tetrahydrocannabivarinic acid; CBL = cannabicyclol; CBC = cannabichromene; THCA = tetrahydrocannabinolic acid; CBCA = cannabichromenic acid; CBT = cannabacitran.

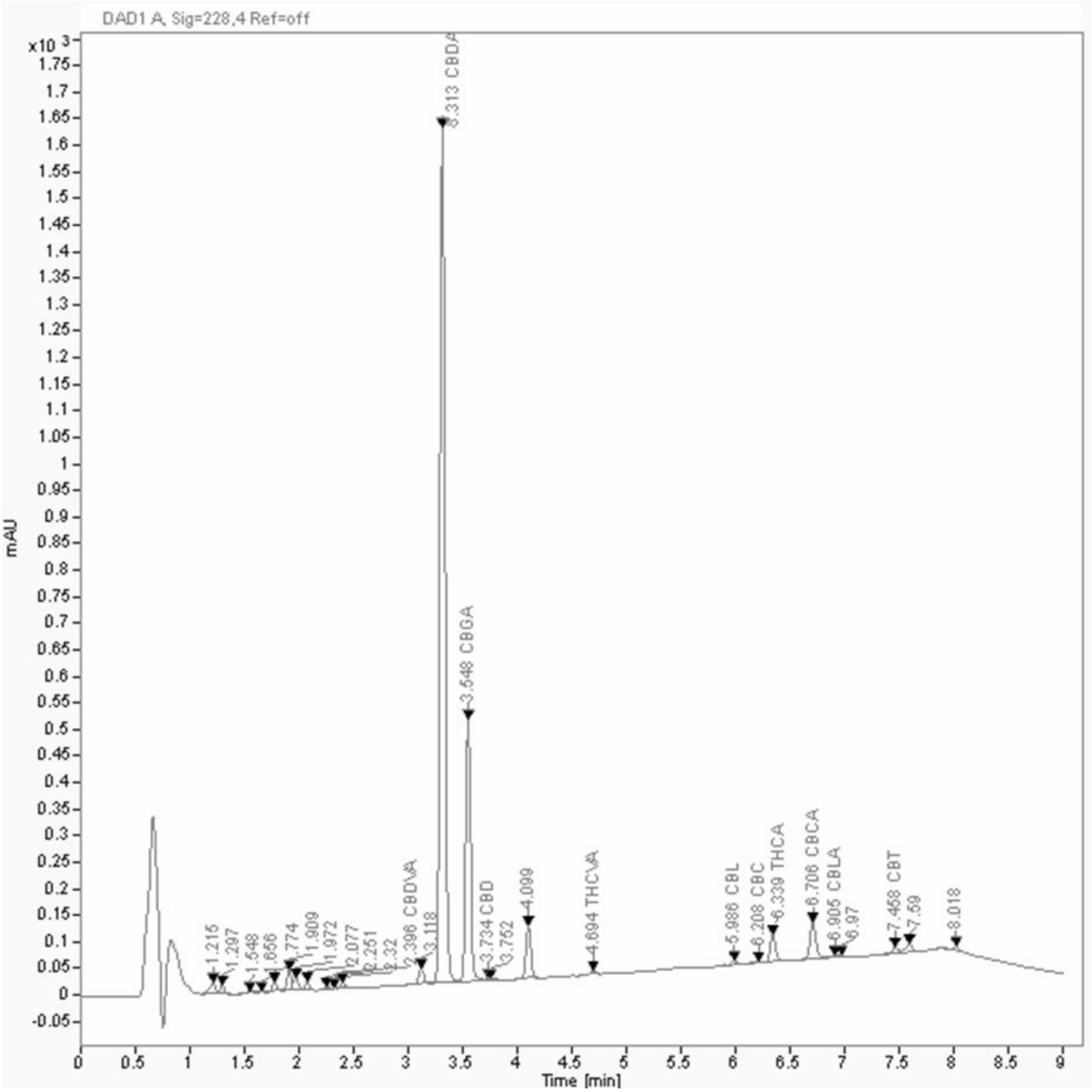

Figure S2. Chromatogram for assessing cannabinoid content in *C. sativa* from individual #3 from the TE population that has a CBDAS:$THCAS_0$ genotype.

Table S2. Percent area report for assessing cannabinoid content in *C. sativa* from individual #3 from the TE population that has a CBDAS:$THCAS_0$ genotype.

Signal:			DAD1 A, Sig=228,4 Ref=off			
RT [min]	Type[1]	Width [min]	Area	Height	Area%	Name[2]
1.215	BV	0.0599	90.9197	22.1449	1.0951	
1.297	VB	0.0440	47.3615	17.2930	0.5704	
1.548	BV	0.0624	13.1755	3.0477	0.1587	
1.656	VB	0.0428	5.0649	1.9192	0.0610	
1.774	BB	0.0471	55.6232	18.5014	0.6700	
1.909	BV	0.0448	108.7997	38.7109	1.3104	
1.972	VB	0.0460	64.0600	22.0009	0.7716	
2.077	BB	0.0442	41.0208	14.8676	0.4941	
2.251	MF	0.0495	14.2584	4.7971	0.1717	
2.320	MF	0.0452	6.7500	2.4885	0.0813	
2.396	MF	0.0599	43.4937	12.1078	0.5239	CBDVA
3.118	BB	0.0577	112.6679	30.0694	1.3570	
3.313	MF	0.0519	5073.6846	1628.8417	61.1099	CBDA
3.548	MF	0.0521	1569.4281	502.0374	18.9030	CBGA
3.734	MF	0.0551	12.6681	3.8339	0.1526	CBD
3.752	FM	0.0379	7.4890	3.2895	0.0902	
4.099	BB	0.0502	310.2503	99.9040	3.7368	
4.694	MF	0.0616	29.6948	8.0298	0.3577	THCVA
5.986	MM	0.0631	30.2835	8.0020	0.3647	CBL
6.208	BB	0.0509	8.9174	2.8175	0.1074	CBC
6.339	MM	0.0583	181.5733	51.8702	2.1870	THCA
6.706	MF	0.0619	260.9765	70.3159	3.1433	CBCA
6.905	MF	0.1058	27.4187	4.3178	0.3302	CBLA
6.970	FM	0.0696	18.9018	4.5268	0.2277	
7.458	BV	0.0508	38.6828	11.6337	0.4659	CBT
7.590	VB	0.0654	69.5749	15.7872	0.8380	
8.018	MM	0.1046	59.8135	9.5263	0.7204	
		Sum	8302.5528			

[1]Indicates type of integration. BV = baseline-to-valley; VB = valley-to-baseline; BB = baseline-to-baseline; MM = manually integrated; R = re-calculated solvent.

[2]Name of cannabinoid. CBDVA = cannabidivarinic acid; CBDA = cannabidiolic acid; CBGA = cannabigerolic acid; CBD = cannabidiol; THCVA = tetrahydrocannabivarinic acid; CBL = cannabicyclol; CBC = cannabichromene; THCA = tetrahydrocannabinolic acid; CBCA = cannabichromenic acid; CBLA = cannabicyclolic acid; CBT = cannabacitran.

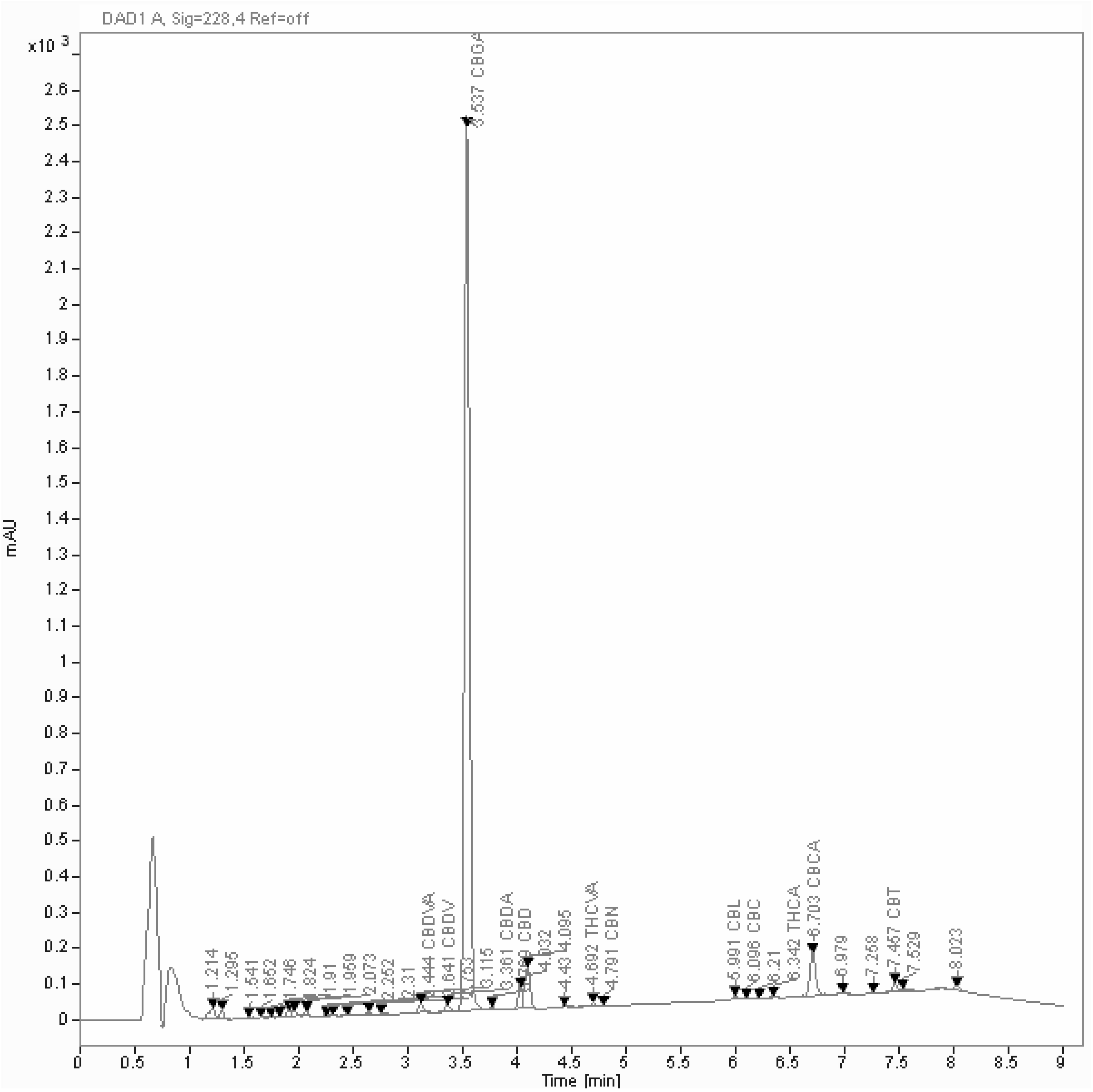

Figure S3. Chromatogram for assessing cannabinoid content in *C. sativa* from individual #1 from the TE population that has a $THCAS_0$:$THCAS_0$ genotype.

Table S3. Percent area report for assessing cannabinoid content in *C. sativa* from individual #1 from the TE population that has a $THCAS_0$:$THCAS_0$ genotype.

Signal:	DAD1 A, Sig=228,4 Ref=off					
RT [min]	Type[1]	Width [min]	Area	Height	Area%	Name[2]
1.214	BV	0.0589	133.6133	31.8803	1.3159	
1.295	VB	0.0435	73.7076	27.2914	0.7259	
1.541	BV	0.0492	16.3282	5.1221	0.1608	
1.652	VB	0.0506	10.8643	3.4592	0.1070	
1.746	BV	0.0468	5.1882	1.7395	0.0511	
1.824	VB	0.0381	5.2131	2.1621	0.0513	
1.910	BV	0.0432	54.7535	19.2449	0.5392	
1.959	VB	0.0440	45.7098	15.6785	0.4502	
2.073	BB	0.0456	43.1513	14.9985	0.4250	
2.252	BV	0.0401	5.0371	1.9528	0.0496	
2.310	VB	0.0466	10.0525	3.2087	0.0990	
2.444	MF	0.0528	4.8780	1.5400	0.0480	CBDVA
2.641	MF	0.0482	23.5305	8.1347	0.2317	CBDV
2.753	FM	0.0621	6.5656	1.7627	0.0647	
3.115	MM	0.0577	95.0426	27.4710	0.9360	
3.361	BB	0.0576	66.3415	18.6252	0.6534	CBDA
3.537	MF	0.0542	8113.7163	2494.8892	79.9084	CBGA
3.769	FM	0.0549	30.5106	9.2692	0.3005	CBD
4.032	BV	0.0448	174.4147	62.0235	1.7177	
4.095	VB	0.0495	382.8356	119.0690	3.7704	
4.430	MM	0.0604	15.5331	4.2882	0.1530	
4.692	MF	0.0614	41.2832	11.2072	0.4066	THCVA
4.791	FM	0.0501	3.5714	1.1881	0.0352	CBN
5.991	BV	0.0582	43.1817	11.3994	0.4253	CBL
6.096	VB	0.0499	11.6940	3.6001	0.1152	CBC
6.210	BB	0.0467	5.4996	1.8479	0.0542	
6.342	BB	0.0535	19.5493	5.7756	0.1925	THCA
6.703	MF	0.0622	455.7289	122.0517	4.4883	CBCA
6.979	FM	0.1200	49.6001	6.8911	0.4885	
7.258	MM	0.0489	7.9454	2.7062	0.0783	
7.457	BV	0.0528	85.1122	25.6035	0.8382	CBT
7.529	VV R	0.0542	27.7920	7.6768	0.2737	
8.023	MM	0.1098	85.8264	13.0284	0.8453	
		Sum	10153.7719			

[1]Indicates type of integration. BV = baseline-to-valley; VB = valley-to-baseline; BB = baseline-to-baseline; MM = manually integrated; R = re-calculated solvent.

[2]Name of cannabinoid. CBDVA = cannabidivarinic acid; CBDV=cannabidivarin; CBDA = cannabidiolic acid; CBGA = cannabigerolic acid; CBD = cannabidiol; THCVA = tetrahydrocannabivarinic acid; CBN=cannabinol; CBL =

cannabicyclol; CBC = cannabichromene; THCA = tetrahydrocannabinolic acid; CBCA = cannabichromenic acid; CBT = cannabacitran.

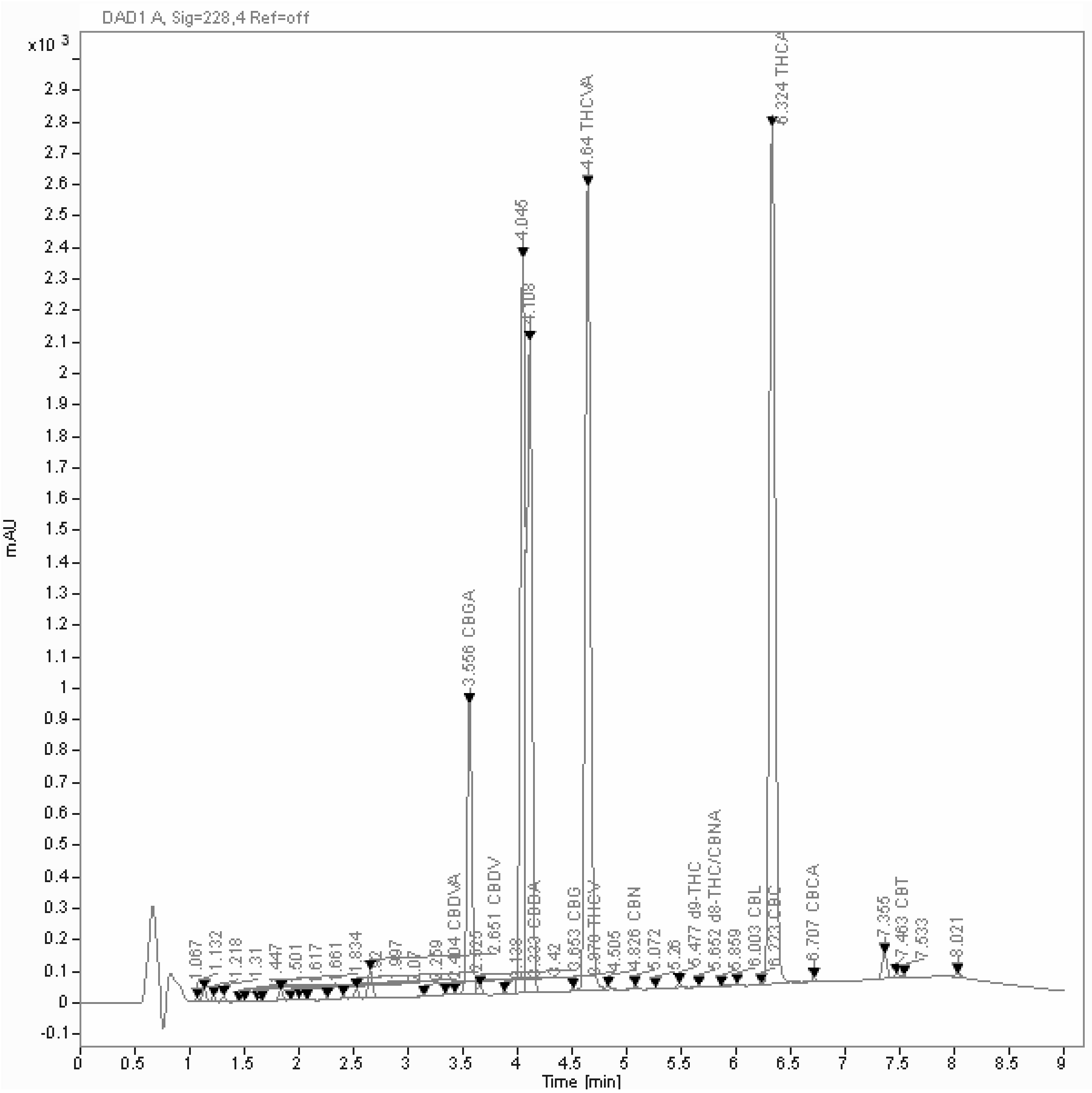

Figure S4. Chromatogram for assessing cannabinoid content in *C. sativa* from individual #32 from the FH population that has a THCAS$_{Wt}$:THCAS$_{Wt}$ genotype.

Table S4. Percent area report for assessing cannabinoid content in *C. sativa* from individual #32 from the FH population that has a $THCAS_{Wt}$:$THCAS_{Wt}$ genotype.

Signal:			DAD1 A, Sig=228,4 Ref=off			
RT [min]	Type[1]	Width [min]	Area	Height	Area%	Name[2]
1.067	BV	0.0374	27.1674	11.5725	0.0715	
1.132	VB	0.0414	101.0796	40.1730	0.2659	
1.218	BB	0.0409	45.1981	18.2489	0.1189	
1.310	BB	0.0435	57.1589	21.1454	0.1504	
1.447	BV	0.0448	11.3595	3.8093	0.0299	
1.501	VB	0.0417	12.8572	4.7307	0.0338	
1.617	BV	0.0384	10.8214	4.1587	0.0285	
1.661	VB	0.0568	18.0056	4.6882	0.0474	
1.834	BB	0.0454	95.6883	33.4661	0.2517	
1.920	BV	0.0367	6.1568	2.6924	0.0162	
1.997	VV	0.0505	26.8502	8.1264	0.0706	
2.070	VB	0.0543	10.8546	2.8594	0.0286	
2.259	BB	0.0447	22.2340	7.9430	0.0585	
2.404	BV	0.0490	40.8248	12.8668	0.1074	CBDVA
2.525	VB	0.0493	107.6923	33.7036	0.2833	
2.651	BB	0.0477	281.4800	92.0647	0.7406	CBDV
3.138	MM	0.0687	21.1928	5.1415	0.0558	
3.333	BV	0.0540	28.0652	8.1821	0.0738	CBDA
3.420	VB	0.0481	22.4886	6.8952	0.0592	
3.556	MF	0.0515	2900.8474	938.6758	7.6319	CBGA
3.653	FM	0.0460	79.2105	28.6762	0.2084	CBG
3.878	BB	0.0471	26.5863	8.8397	0.0699	THCV
4.045	BV	0.0471	7522.5083	2365.5620	19.7911	
4.108	VB	0.0482	6429.9697	2074.5356	16.9167	
4.505	MF	0.0598	46.3475	12.9112	0.1219	
4.640	FM	0.0587	9105.5146	2584.1250	23.9559	THCVA
4.826	FM	0.0609	49.0040	13.4037	0.1289	CBN
5.072	MM	0.0570	39.4379	11.5262	0.1038	
5.260	BB	0.0517	16.1198	4.9907	0.0424	
5.477	MM	0.0576	54.5517	15.7805	0.1435	d9-THC
5.652	MM	0.0610	20.3199	5.5528	0.0535	d8-THC/CBNA
5.859	MF	0.0560	9.6876	2.8850	0.0255	
6.003	FM	0.0690	31.7086	7.6600	0.0834	CBL
6.223	MF	0.0441	14.2160	5.3701	0.0374	CBC
6.324	FM	0.0618	10231.7314	2760.2231	26.9189	THCA
6.707	BB	0.0563	53.4617	14.7301	0.1407	CBCA
7.355	MF	0.0545	273.0882	83.5550	0.7185	
7.463	MF	0.0558	50.6248	15.1312	0.1332	CBT
7.533	FM	0.0519	33.3454	10.7050	0.0877	
8.021	MM	0.0888	74.0522	13.8925	0.1948	
		Sum	38009.5087			

[1]Indicates type of integration. BV = baseline-to-valley; VB = valley-to-baseline; BB = baseline-to-baseline; MM = manually integrated; R = re-calculated solvent.

[2]Name of cannabinoid. CBDVA = cannabidivarinic acid; CBDV=cannabidivarin; CBDA = cannabidiolic acid; CBGA = cannabigerolic acid; CBG = cannabigerol; THCV = tetrahydrocannabivarin; THCVA = tetrahydrocannabivarinic acid; CBN=cannabinol; d9-THC = delta-9-tetrahydrocannabinol; d8-THC/CBNA = delta-8-tetrahydrocannabinol/cannabinolic acid; CBL = cannabicyclol; CBC = cannabichromene; THCA = tetrahydrocannabinolic acid; CBCA = cannabichromenic acid; CBT = cannabacitran.

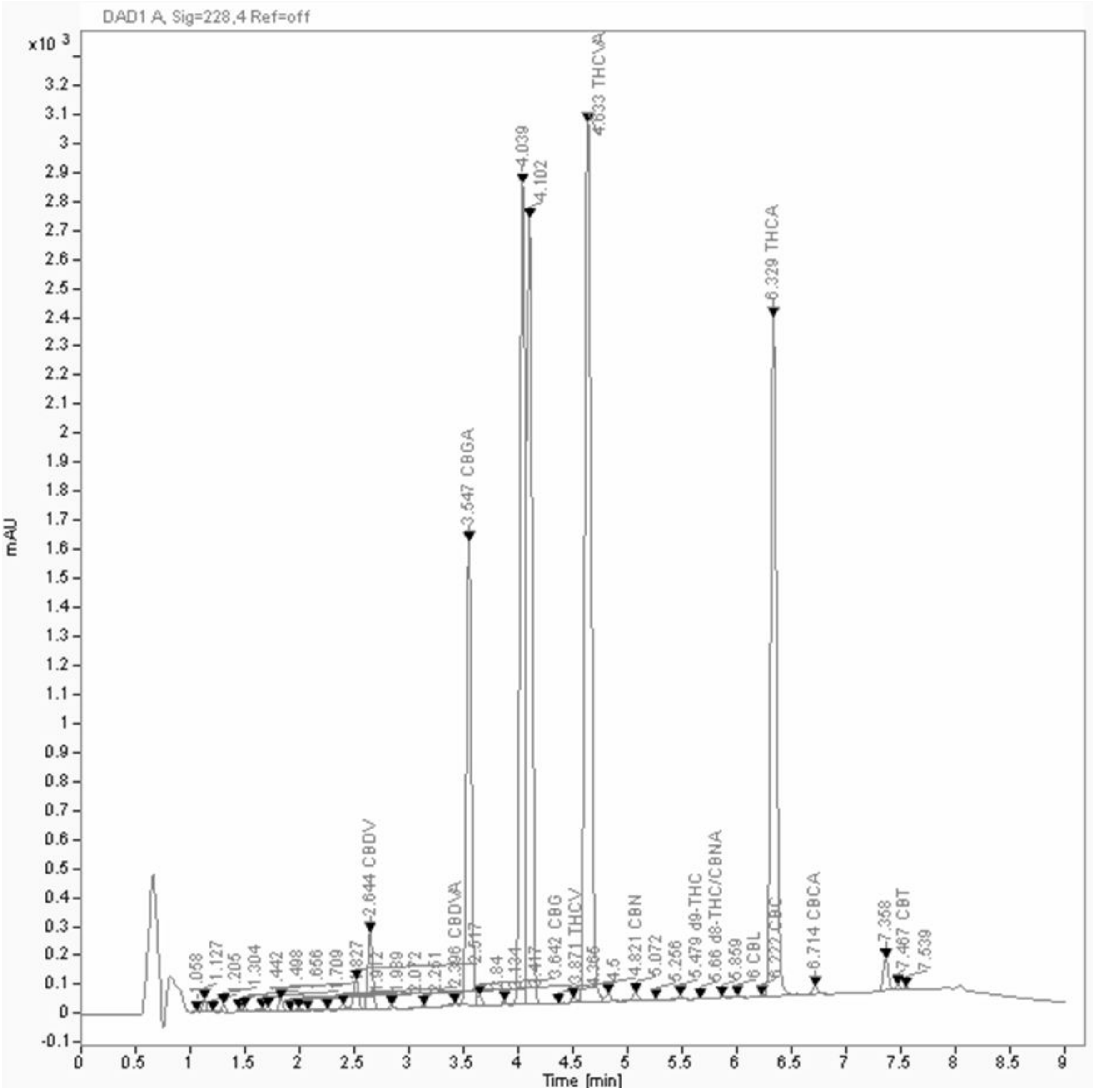

Figure S5. Chromatogram for assessing cannabinoid content in *C. sativa* from individual #1 from the FH population that has a $THCAS_{Wt}$:$THCAS_0$ genotype.

Table S5. Percent area report for assessing cannabinoid content in *C. sativa* from individual #1 from the FH population that has a $THCAS_{Wt}$:$THCAS_0$ genotype.

Signal:			DAD1 A, Sig=228,4 Ref=off			
RT [min]	Type[1]	Width [min]	Area	Height	Area%	Name[2]
1.058	BV E	0.0349	12.9571	6.0810	0.0277	
1.127	VV R	0.0428	126.2816	47.8684	0.2696	
1.205	VB E	0.0436	16.6116	6.1299	0.0355	
1.304	BB	0.0428	81.3789	30.8354	0.1737	
1.442	BV	0.0419	32.6334	11.9163	0.0697	
1.498	VB	0.0436	44.4739	16.4375	0.0950	
1.656	BV	0.0599	47.2062	11.0410	0.1008	
1.709	VB	0.0425	36.0905	13.8047	0.0771	
1.827	BB	0.0445	123.2571	44.2677	0.2632	
1.912	BV	0.0376	10.2405	4.3347	0.0219	
1.989	VV	0.0492	29.1732	9.1496	0.0623	
2.072	VB	0.0739	21.9521	4.0081	0.0469	
2.251	BB	0.0437	20.9542	7.7183	0.0447	
2.396	BV	0.0479	67.2902	21.8499	0.1437	CBDVA
2.517	VB	0.0491	332.5999	104.7258	0.7101	
2.644	BB	0.0492	809.8193	268.7694	1.7290	CBDV
2.840	MM	0.0489	40.8434	13.9193	0.0872	
3.134	MM	0.0616	38.5994	10.4494	0.0824	
3.417	BB	0.0896	52.6103	7.9048	0.1123	
3.547	MF	0.0517	5035.5542	1624.8540	10.7511	CBGA
3.642	FM	0.0434	96.4417	37.0065	0.2059	CBG
3.871	BB	0.0492	50.1113	16.5922	0.1070	THCV
4.039	BV	0.0501	9327.2012	2858.8354	19.9139	
4.102	VB	0.0498	8851.8369	2730.6721	18.8990	
4.365	BB	0.0538	5.2135	1.6100	0.0111	
4.500	MF	0.0594	71.5180	20.0823	0.1527	
4.633	MF	0.0654	12041.5566	3068.4099	25.7091	THCVA
4.821	FM	0.0603	104.1017	28.7863	0.2223	CBN
5.072	BB	0.0580	111.5604	29.6122	0.2382	
5.256	BB	0.0512	19.8952	6.2471	0.0425	
5.479	MM	0.0607	53.4070	14.6641	0.1140	d9-THC
5.660	MM	0.0534	12.9791	4.0495	0.0277	d8-THC/CBNA
5.859	MF	0.0513	23.0474	7.4888	0.0492	
6.000	FM	0.0650	31.2713	8.0191	0.0668	CBL
6.222	MF	0.0501	19.2774	6.4171	0.0412	CBC
6.329	FM	0.0602	8504.8252	2353.5413	18.1581	THCA
6.714	BB	0.0594	105.1877	28.2940	0.2246	CBCA
7.358	BB	0.0510	350.6801	110.5148	0.7487	
7.467	BV	0.0458	49.8782	17.2350	0.1065	CBT
7.539	VB	0.0463	27.1944	9.2600	0.0581	
		Sum	46837.7110			

[1]Indicates type of integration. BV = baseline-to-valley; VB = valley-to-baseline; BB = baseline-to-baseline; MM = manually integrated; R = re-calculated solvent.

[2]Name of cannabinoid. CBDVA = cannabidivarinic acid; CBDV=cannabidivarin; CBGA = cannabigerolic acid; CBG = cannabigerol; THCV = tetrahydrocannabivarin; THCVA = tetrahydrocannabivarinic acid; CBN=cannabinol; d9-THC = delta-9-tetrahydrocannabinol; d8-THC/CBNA = delta-8-tetrahydrocannabinol/cannabinolic acid; CBL = cannabicyclol; CBC = cannabichromene; THCA = tetrahydrocannabinolic acid; CBCA = cannabichromenic acid; CBT = cannabacitran.

Figure S6. Chromatogram for assessing cannabinoid content in *C. sativa* from individual #13 from the FH population that has a $THCAS_0$:$THCAS_0$ genotype.

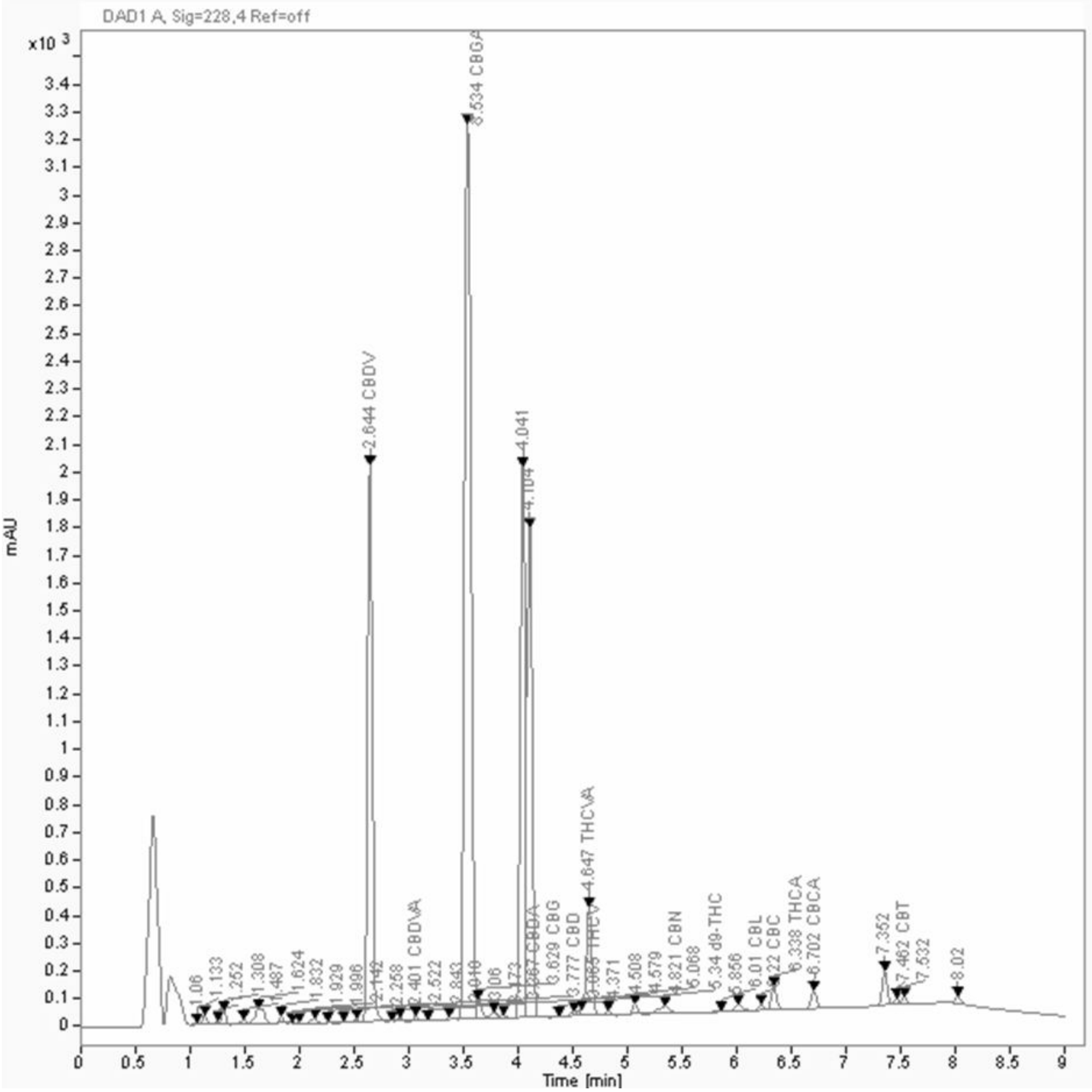

Table S6. Percent area report for assessing cannabinoid content in *C. sativa* from individual #13 from the FH population that has a $THCAS_0$:$THCAS_0$ genotype.

Signal:	DAD1 A, Sig=228,4 Ref=off					
RT [min]	Type[1]	Width [min]	Area	Height	Area%	Name[2]
1.060	BB	0.0350	11.8419	5.5306	0.0326	
1.133	BV	0.0404	89.2423	34.2561	0.2457	
1.252	VV	0.0451	48.8856	14.5142	0.1346	
1.308	VB	0.0458	152.2942	52.5008	0.4193	
1.487	BV	0.0677	75.2178	16.9674	0.2071	
1.624	VB	0.0830	362.3113	56.3612	0.9975	
1.832	BB	0.0431	79.0103	27.8720	0.2175	
1.929	BV	0.0469	15.5379	5.1914	0.0428	
1.996	VB	0.0413	15.1818	5.6678	0.0418	
2.142	BB	0.0670	91.9771	18.8215	0.2532	
2.258	BB	0.0432	21.2930	7.9697	0.0586	
2.401	BV	0.0560	28.7220	7.9851	0.0791	CBDVA
2.522	VB	0.0460	38.3463	13.1459	0.1056	
2.644	BB	0.0469	6079.7920	2030.7074	16.7392	CBDV
2.843	BV E	0.0390	6.0608	2.4387	0.0167	
2.918	VB R	0.0471	41.6373	13.8232	0.1146	
3.060	BV R	0.0484	51.0263	17.3291	0.1405	
3.173	VB E	0.0500	5.3694	1.6489	0.0148	
3.367	BB	0.0585	36.8474	9.2543	0.1014	CBDA
3.534	MF	0.0693	13537.4883	3254.1270	37.2721	CBGA
3.629	FM	0.0402	165.1014	68.3845	0.4546	CBG
3.777	FM	0.0658	95.0379	24.0623	0.2617	CBD
3.865	FM	0.0625	47.5599	12.6794	0.1309	THCV
4.041	BV	0.0486	6295.2959	2008.5109	17.3325	
4.104	VB	0.0481	5509.0859	1781.6635	15.1679	
4.371	BB	0.0515	7.9287	2.4692	0.0218	
4.508	MF	0.0616	61.6268	16.6769	0.1697	
4.579	FM	0.0387	52.1798	22.4556	0.1437	
4.647	FM	0.0575	1364.2090	395.7478	3.7560	THCVA
4.821	BB	0.0552	54.8831	16.3156	0.1511	CBN
5.068	BV	0.0592	153.7773	39.6592	0.4234	
5.340	VB	0.1150	237.1593	27.9480	0.6530	d9-THC
5.856	MF	0.0567	16.9699	4.9841	0.0467	
6.010	FM	0.0925	146.3149	26.3501	0.4028	CBL
6.220	BV	0.0504	71.7938	23.0360	0.1977	CBC
6.338	VB	0.0568	316.1783	86.2114	0.8705	THCA
6.702	BB	0.0567	239.8205	65.5010	0.6603	CBCA
7.352	MF	0.0538	401.4206	124.2922	1.1052	
7.462	MF	0.0549	78.6226	23.8612	0.2165	CBT
7.532	FM	0.0533	89.6795	28.0461	0.2469	
8.020	MM	0.0776	128.0126	27.4865	0.3525	

Sum	36320.7407

[1]Indicates type of integration. BV = baseline-to-valley; VB = valley-to-baseline; BB = baseline-to-baseline; MM = manually integrated; R = re-calculated solvent.

[2]Name of cannabinoid. CBDVA = cannabidivarinic acid; CBDV=cannabidivarin; CBDA = cannabidiolic acid; CBGA = cannabigerolic acid; CBG = cannabigerol; CBD = cannabidiol; THCV = tetrahydrocannabivarin; THCVA = tetrahydrocannabivarinic acid; CBN=cannabinol; d9-THC = delta-9-tetrahydrocannabinol; CBL = cannabicyclol; CBC = cannabichromene; THCA = tetrahydrocannabinolic acid; CBCA = cannabichromenic acid; CBT = cannabacitran.

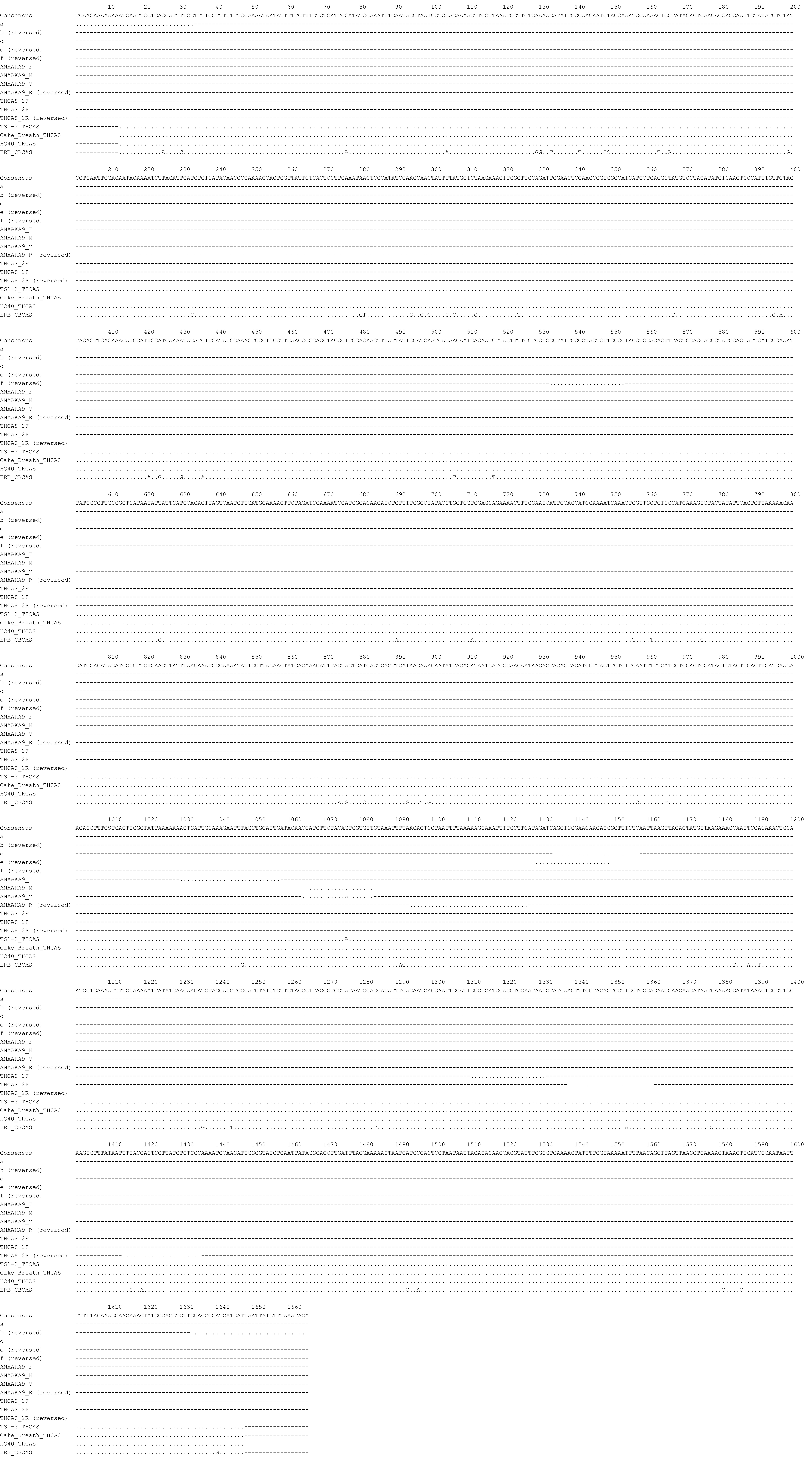
Consensus
a
b (reversed)
d
e (reversed)
f (reversed)
ANAAKA9_F
ANAAKA9_M
ANAAKA9_V
ANAAKA9_R (reversed)
THCAS_2F
THCAS_2P
THCAS_2R (reversed)
TS1-3_THCAS
Cake_Breath_THCAS
HO40_THCAS
ERB_CBCAS

Figure S7. Sequence alignment containing nucleotide sequences from several *C. sativa* strains and primer and probe sequences used in this paper. A consensus sequence is provided at the top. Dots represent sequence homology, SNPs are indicated with the alternative nucleotide. Dashes indicate that the sequence data for that entry in the alignment do not span that particular region. Some primer sequences target the antisense strand to the consensus sequence reported and are therefore reported in the 3′ to 5′ direction and labeled as "reversed."

```
                            10        20        30        40        50        60        70        80        90       100       110       120       130       140       150
Consensus           ATGAAGTRCTCAACATTCTCCTTTTGGTTTGTTTGCAAGATAATATTTTTCTTTTTCTCATTCAATATCCAAACTTCCATTGCTAATCCTCGAGAAAACTTCCTTAAATGCTTCTCGCAATATATTCCCAATAATGCAACAAATCTAAAA
CBDAS_6F            ------------------------------------------------------------------------------------------------------------------------------------------------------
CBDAS_6P            ------------------------------------------------------------------------------------------------------------------------------------------------------
CBDAS_6R (reversed) ------------------------------------------------------------------------------------------------------------------------------------------------------
ERB_CBDAS           ......................................................................................................................................................

                           160       170       180       190       200       210       220       230       240       250       260       270       280       290       300
Consensus           CTCGTATACACTCAAAACAACCCATTGTATATGTCTGTCCTAAATTCGACAATACACAATCTTAGATTCACCTCTGACACAACCCCAAAACCACTTGTTATCGTCACTCCTTCACATGTCTCTCATATCCAAGGCACTATTCTATGCTCC
CBDAS_6F            ------------------------------------------------------------------------------------------------------------------------------------------------------
CBDAS_6P            ------------------------------------------------------------------------------------------------------------------------------------------------------
CBDAS_6R (reversed) ------------------------------------------------------------------------------------------------------------------------------------------------------
ERB_CBDAS           ......................................................................................................................................................

                           310       320       330       340       350       360       370       380       390       400       410       420       430       440       450
Consensus           AAGAAAGTTGGCTTGCAGATTCGAACTCGAAGTGGTGGTCATGATTCTGAGGGCATGTCCTACATATCTCAAGTCCCATTTGTTATAGTAGACTTGAGAAACATGCGTTCAATCAAAATAGATGTTCATAGCCAAACTGCATGGGTTGAA
CBDAS_6F            ------------------------------------------------------------------------------------------------------------------------------------------------------
CBDAS_6P            ------------------------------------------------------------------------------------------------------------------------------------------------------
CBDAS_6R (reversed) ------------------------------------------------------------------------------------------------------------------------------------------------------
ERB_CBDAS           ......................................................................................................................................................

                           460       470       480       490       500       510       520       530       540       550       560       570       580       590       600
Consensus           GCCGGAGCTACCCTTGGAGAAGTTTATTATTGGGTTAATGAGAAAAATGAGAATCTTAGTTTGGCKGCTGGGTATTGCCCTACTGTTTGCGCAGGTGGACACTTTGGTGGAGGAGGCTATGGACCATTGATGAGAAACTATGGCCTCGCG
CBDAS_6F            -----------------------------------------------------------------------------------------------------....................-----------------------------
CBDAS_6P            -------------------------------------------------------------------------------------------------------------------------------.......................
CBDAS_6R (reversed) ------------------------------------------------------------------------------------------------------------------------------------------------------
ERB_CBDAS           ......................................................................................................................................................

                           610       620       630       640       650       660       670       680       690       700       710       720       730       740       750
Consensus           GCTGATAATATCATTGATGCACACTTAGTCAACGTTCATGGAAAAGTGCTAGATCGAAAATCTATGGGGGAAGATCTCTTTTGGGCTTTACGTGGTGGTGGAGCAGAAAGCTTCGGAATCATTGTAGCATGGAAAATTAGACTGGTTGCT
CBDAS_6F            ------------------------------------------------------------------------------------------------------------------------------------------------------
CBDAS_6P            .-----------------------------------------------------------------------------------------------------------------------------------------------------
CBDAS_6R (reversed) --------------------......................------------------------------------------------------------------------------------------------------------
ERB_CBDAS           ......................................................................................................................................................

                           760       770       780       790       800       810       820       830       840       850       860       870       880       890       900
Consensus           GTCCCAAAGTCTACTATGTTTAGTGTTAAAAAGATCATGGAGATACATGAGCTTGTCAAGTTAGTTAACAAATGGCAAAATATTGCTTACAAGTATGACAAAGATTTATTACTCATGACTCACTTCATAACTAGGAACATTACAGATAAT
CBDAS_6F            ------------------------------------------------------------------------------------------------------------------------------------------------------
CBDAS_6P            ------------------------------------------------------------------------------------------------------------------------------------------------------
CBDAS_6R (reversed) ------------------------------------------------------------------------------------------------------------------------------------------------------
ERB_CBDAS           ......................................................................................................................................................

                           910       920       930       940       950       960       970       980       990      1000      1010      1020      1030      1040      1050
Consensus           CAAGGGAAGAATAAGACAGCAATACACACTTACTTCTCTTCAGTTTTCCTTGGTGGAGTGGATAGTCTAGTCGACTTGATGAACAAGAGTTTTCCTGAGTTGGGTATTAAAAAAACGGATTGCAGACAATTGAGCTGGATTGATACTATC
CBDAS_6F            ------------------------------------------------------------------------------------------------------------------------------------------------------
CBDAS_6P            ------------------------------------------------------------------------------------------------------------------------------------------------------
CBDAS_6R (reversed) ------------------------------------------------------------------------------------------------------------------------------------------------------
ERB_CBDAS           ......................................................................................................................................................

                          1060      1070      1080      1090      1100      1110      1120      1130      1140      1150      1160      1170      1180      1190      1200
Consensus           ATCTTCTATAGTGGTGTTGTAAATTACGACACTGATAATTTTAACAAGGAAATTTTGCTTGATAGATCCGCTGGGCAGAACGGTGCTTTCAAGATTAAGTTAGACTACGTTAAGAAACCAATTCCAGAATCTGTATTTGTCCAAATTTTG
CBDAS_6F            ------------------------------------------------------------------------------------------------------------------------------------------------------
CBDAS_6P            ------------------------------------------------------------------------------------------------------------------------------------------------------
CBDAS_6R (reversed) ------------------------------------------------------------------------------------------------------------------------------------------------------
ERB_CBDAS           ......................................................................................................................................................

                          1210      1220      1230      1240      1250      1260      1270      1280      1290      1300      1310      1320      1330      1340      1350
Consensus           GAAAAATTATATGAAGAAGATATAGGAGCTGGGATGTATGCGTTGTACCCTTACGGTGGTATAATGGATGAGATTTCWGAATCAGCAATTCCATTCCCTCATCGAGCTGGAATCTTGTATGAGTTATGGTACATATGTAGYTGGGAGAAG
CBDAS_6F            ------------------------------------------------------------------------------------------------------------------------------------------------------
CBDAS_6P            ------------------------------------------------------------------------------------------------------------------------------------------------------
CBDAS_6R (reversed) ------------------------------------------------------------------------------------------------------------------------------------------------------
ERB_CBDAS           ......................................................................................................................................................

                          1360      1370      1380      1390      1400      1410      1420      1430      1440      1450      1460      1470      1480      1490      1500
Consensus           CAAGAAGATAACGAAAAGCATCTAAACTGGATTAGAAATATTTATAACTTCATGACTCCTTATGTGTCCMAAAATCCAAGATTGGCATATCTCAATTATAGAGACCTTGATATAGGAATAAATGATCCCAAGAATCCAAATAATTACACA
CBDAS_6F            ------------------------------------------------------------------------------------------------------------------------------------------------------
CBDAS_6P            ------------------------------------------------------------------------------------------------------------------------------------------------------
CBDAS_6R (reversed) ------------------------------------------------------------------------------------------------------------------------------------------------------
ERB_CBDAS           ......................................................................................................................................................

                          1510      1520      1530      1540      1550      1560      1570      1580      1590      1600      1610      1620      1630
Consensus           CAAGCACGTATTTGGGGTGAGAAGTATTTTGGTAAAAATTTTGACAGGCTAGTAAAAGTGAAAACCCTGGTTGATCCCAATAAYTTTTTYAGAAACGAACAAAGCATCCCACCTCTTCCACGGCATCRTCATTAA
CBDAS_6F            ---------------------------------------------------------------------------------------------------------------------------------------
CBDAS_6P            ---------------------------------------------------------------------------------------------------------------------------------------
CBDAS_6R (reversed) ---------------------------------------------------------------------------------------------------------------------------------------
ERB_CBDAS           .......................................................................................................................................
```

Figure S8. Sequence alignment containing nucleotide sequences from *C. sativa* strain ERB and primer and probe sequences used in this paper. A consensus sequence is provided at the top. Dots represent sequence homology, SNPs are indicated with the alternative nucleotide. Dashes indicate that the sequence data for that entry in the alignment do not span that particular region. Primers which target the antisense strand to the consensus sequence reported are reported in the 3′ to 5′ direction and labeled as "reversed."
